# Gut microbiota, intestinal permeability, and systemic inflammation: a narrative review

**DOI:** 10.1007/s11739-023-03374-w

**Published:** 2023-07-28

**Authors:** Federica Di Vincenzo, Angelo Del Gaudio, Valentina Petito, Loris Riccardo Lopetuso, Franco Scaldaferri

**Affiliations:** 1 UOS Malattie Infiammatorie Croniche Intestinali, Centro Malattie Apparato Digerente (CeMAD), Dipartimento di Scienze Mediche e Chirurgiche, Fondazione Policlinico Universitario “A. Gemelli” IRCCS, L.go A. Gemelli 8, Rome, Italy; 2 Dipartimento di Medicina e Chirurgia Traslazionale, Università Cattolica del Sacro Cuore, L.go F. Vito 1, Rome, Italy

**Keywords:** Intestinal barrier, Gut microbiota, Intestinal permeability, Systemic inflammation, Metabolic disease

## Abstract

The intestine is the largest interface between the internal body and the external environment. The intestinal barrier is a dynamic system influenced by the composition of the intestinal microbiome and the activity of intercellular connections, regulated by hormones, dietary components, inflammatory mediators, and the enteric nervous system (ENS). Over the years, it has become increasingly evident that maintaining a stable intestinal barrier is crucial to prevent various potentially harmful substances and pathogens from entering the internal environment. Disruption of the barrier is referred to as 'leaky gut' or leaky gut wall syndrome and seems to be characterized by the release of bacterial metabolites and endotoxins, such as lipopolysaccharide (LPS), into the circulation. This condition, mainly caused by bacterial infections, oxidative stress, high-fat diet, exposure to alcohol or chronic allergens, and dysbiosis, appear to be highly connected with the development and/or progression of several metabolic and autoimmune systemic diseases, including obesity, non-alcoholic fatty liver disease (NAFLD), neurodegeneration, cardiovascular disease, inflammatory bowel disease, and type 1 diabetes mellitus (T1D). In this review, starting from a description of the mechanisms that enable barrier homeostasis and analyzing the relationship between this complex ecosystem and various pathological conditions, we explore the role of the gut barrier in driving systemic inflammation, also shedding light on current and future therapeutic interventions.

## Introduction

The intestine is the most extended interface between the internal body and the external environment. The maintenance of a stable intestinal barrier is crucial to prevent luminal substances and pathogens from entering the internal environment. The intestinal homeostasis, which is the healthy and balanced state of the intestine, is determined by the intestinal epithelium, the gut microbiome, and the host immune system. This functional unit strictly depends on the integrity of the gut epithelium, supported by junctional proteins, such as tight junctions (TJs), desmosomes, and adherent junctions, which form a physical barrier and connect adjacent epithelial cells, together with the lamina propria.

The gut barrier is a dynamic system influenced by the composition of the intestinal microbiome and the activity of intercellular connections, regulated by hormones, dietary components, inflammatory mediators, and the enteric nervous system (ENS). The ENS is also called the “second brain”, since it can regulate intestinal secretion and motility independently from the brain itself [1].

Under physiological conditions, the intestinal barrier must ensure the right balance between the selective permeability of dietary nutrients from the intestinal lumen to the systemic circulation and internal environment, and the protection of the body from the penetration of pathogens and harmful components of the external environment. Selective absorption of nutrients occurs through intercellular or transcellular transport, while harmful and waste substances are removed from the gastrointestinal tract through the feces.

Violation of the integrity of the intestinal barrier and its improper functioning can result in the uncontrolled passage of bacterial components, products of bacterial metabolism, and harmful substances, thus leading to systemic inflammation.

**Fig. 1:**
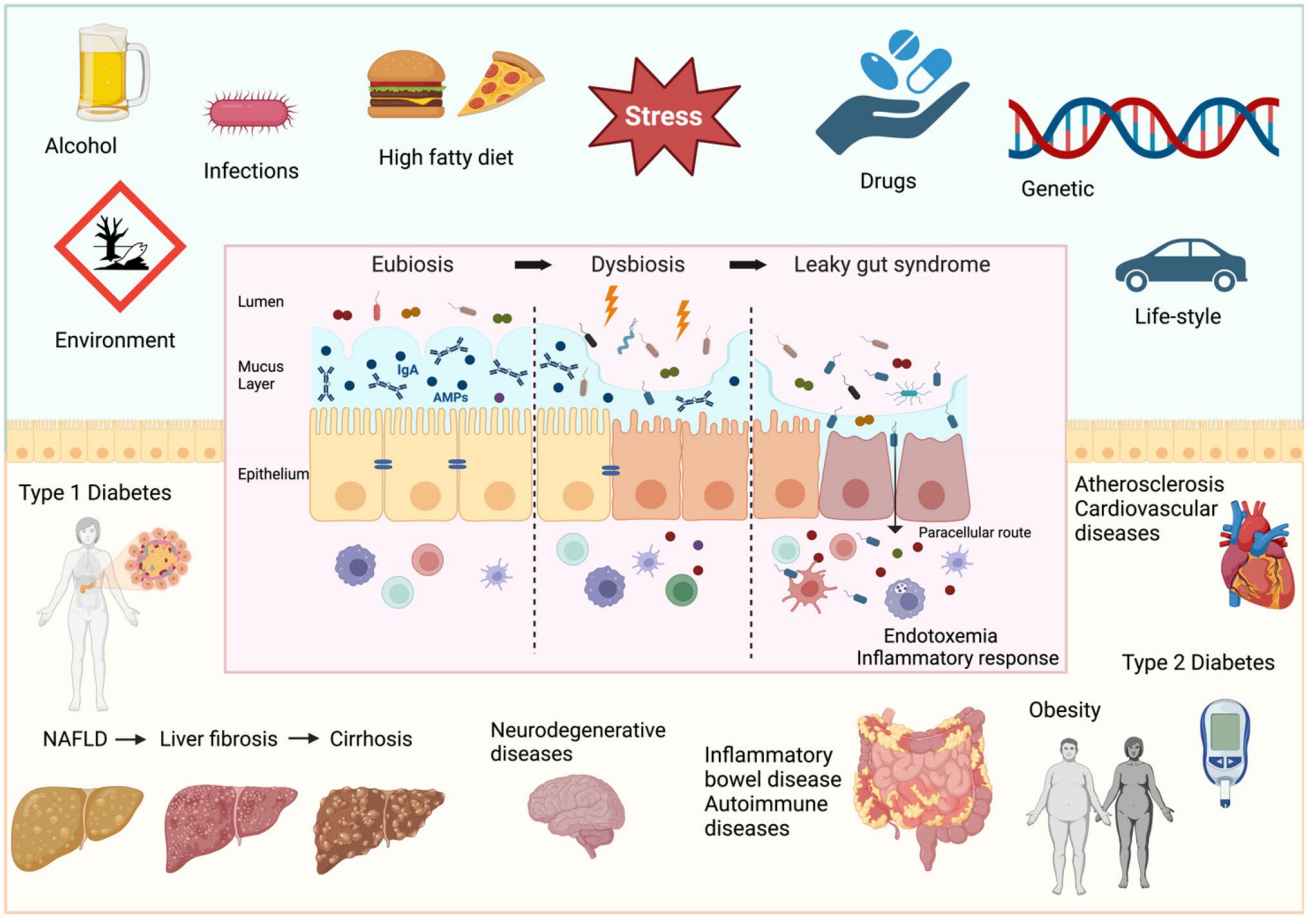
Factors determining intestinal barrier impairment and consequent systemic diseases

The impairment of the intestinal barrier is referred to as “leaky gut” or intestinal wall leakage syndrome. This condition, mainly caused by bacterial infections, oxidative stress, alcohol or chronic allergen exposure, and dysbiosis, leads to the development of several pathologic conditions, including obesity, non-alcoholic fatty liver disease (NAFLD), non-alcoholic steatohepatitis (NASH), liver cirrhosis, neurodegeneration, cardiovascular diseases, inflammatory bowel disease, celiac disease, irritable bowel syndrome, type 1 diabetes mellitus (T1D), and several autoimmune conditions (Fig. 1) [2].

Therefore, maintaining the integrity of the intestinal barrier has a comprehensive impact on human health, so several studies have been conducted to understand the mechanisms involved in improving the integrity of the gut barrier.

Here, we explore the role of the intestinal barrier in driving systemic inflammation, touching upon on the consequent pathological conditions and the potential gut barrier-targeted therapeutic opportunities following these findings.

### The intestinal barrier

The intestinal barrier is a dynamic entity interfacing with and responding to several stimuli. It is composed of multiple elements. The intestinal lumen is protected by pathogens bacteria by bile, gastric acids, pancreatic juice, and commensal bacteria with their metabolites and antimicrobial substances. Going from the inside to the outside, we find a microclimate consisting of the unstirred water layer, glycocalyx, and mucus layer which prevent bacterial adhesion by physical barrier provided by the glycocalyx and mucus and by the secretion of antimicrobial proteins (AMPs) and immunoglobulin A (IgA). The middle layer is composed of intestinal epithelial cells (IECs), while the inner layer is inhabited by immune cells of innate and adaptative immunity [3].

Interestingly, Spadoni et al. identified the existence of a gut–vascular barrier (GVB) that controls the type of molecules and antigens that are translocated across the blood endothelial cells, allowing the passage of small molecules and nutrients, but not bacteria or large bacterial molecules. Notably, they have demonstrated that the GVB can be disrupted by *Salmonella* infection and that is modified in celiac patients with elevated serum transaminases [4].

### The mucus layer

The mucus is a substance composed of water for over 98% and of glycoproteins, such as mucine 2 (MUC2), MUC6, and MUC5A in the stomach and MUC2 in the small and large intestine, secreted by goblet cells. Intestinal epithelial cells (IECs) also express transmembrane mucins, such as MUC12, MUC1, and MUC3 attached to the apical surface and forming, together with the glycolipids, the glycocalyx [5].

The mucus layer is composed of two components: an inner firmly adherent layer with sparse bacteria and protective secreted peptides with antibacterial functions (e.g., lysozyme and defensins); and a thicker and loosely outer layer rich of bacteria and bacterial products. The mucus layer in the colon is thicker, compared with the small intestine where pore size ranges from 4 to 5 Å at the villus tip to over 20 Å at the base of the crypt [5]. For this reason, in the small bowel, enterocytes, Paneth cells, and immune cells secrete antimicrobial products for host defense [5]. The mucus layer has a mutualistic relationship with the gut microbiota, influencing each other. Germ free (GF) mice have a different mucus layer than conventionally raised mice: in GF mice, the number of filled goblet cells is lower, and the small intestinal mucus is strictly anchored to the goblet cells and cannot be aspirated off. A study by Petersson et al. demonstrated that mice-lacking colonic mucus (MUC2 -/-) were hypersensitive to the development of DSS-induced colitis. Moreover, they showed that microbial products like LPS and peptidoglycan stimulate mucus secretion, thus restoring mucus homeostasis [6].

### The epithelium

The intestinal epithelium is composed of five different types of cells: enterocytes, goblet cells, enteroendocrine cell, Paneth cells, and microfold cells. All these cells are renewed by the same pool of stem cells residing in the intestinal crypts [3].

The intestinal epithelium is impermeable to water and hydrophilic solutes. Therefore, any molecule and nutrient can be absorbed only through specific transport routes, represented by the transcellular route, including aqueous pores, endocytosis, and active carrier-mediated absorption for nutrients; and the paracellular route for hydrophilic molecules and ions. From the apical to the basal domains of enterocytes, there are four sets of intercellular junctions represented by: tight junctions (TJs) [zonula occludens (ZO)], adherens junctions (zonula adherens), desmosomes, and gap junctions. Together, they form the apical junctional complex, regulating epithelial barrier function and intercellular transport.

Tight junctions play a critical role in the building of the epithelial barrier and in the regulation of epithelial polarity.

The TJs are composed of three groups of transmembrane proteins which interact with cytoskeletal actomyosin ring: claudin family, Marvel domain-containing proteins, and immunoglobulin superfamily [2].

Generally, it is recognized that there are three distinct paracellular epithelial permeability pathways: “leak” and “pore” pathways, both regulated by TJs, which define intestinal permeability under condition of homeostasis; and an “unrestricted” pathway, associated with apoptotic leaks in pathological states and independent of TJs. It provides, in the presence of erosions or ulcers, access of luminal antigens and bacteria to the lamina propria [2].

## The gut microbiota

The human gut is inhabited by several community of microorganisms, of whom approximately 10^13^ bacterial cells were globally referred to as “gut microbiota”. The gut microbiota is composed of more than 250 species of viruses, fungi, bacteria, and archaea, and it is a dynamic system that changes throughout the human life. The relationship between gut microbiota and host is highly mutualistic, since the latter plays a crucial role in several physiological and pathological pathways of human life [7]. Human gut microbiota is composed of five predominantly phyla of bacteria: Firmicutes (60 to 80%), which includes the classes of *Clostridia*, *Bacilli* and *Negativicutes*, the Bacteroidetes (20 to 40%, including *Flavobacteria*, *Bacteroidia*, *Sphingobacteria* and *Cytophagia*, the Verrucomicrobia, the Actinobacteria and a lesser extent of Proteobacteria; and one Archea phyla, the Euryarchaeota [7].

It exerts a role in the digestion of common polysaccharides, like glycosaminoglycan degradation and short-chain fatty acids’ (SCFAs) production, through the production of different enzymes. Moreover, it is a source of essential amino acids and vitamins like vitamin K, thiamine, folate, biotin, riboflavin, and pantothenic acid. Furthermore, gut microbiota is involved in the maintaining of the integrity of the intestinal epithelial barrier, in the protection against exogenous pathogens, in the maturation of the host gut immune system, and in the metabolism of xenobiotics [8]. The microbiota’s complex functions include also influences of distant organs, outside the intestinal tracts; indeed, it behaves as an endocrine organ, influencing satiety regulation, hormonal regulation, human mood, and behavior. The reciprocal interaction between intestinal microbiome and brain is called the “gut-brain axis”.

### From eubiosis to dysbiosis

A hot topic for microbiota research is represented by the definition of Eubiosis or “healthy microbiota”. It can be considered as the balance of the intestinal microbial ecosystem with beneficial effects for the whole human body. Overall, we can assume that healthy gut microbial communities are characterized by high taxa diversity, high microbial gene richness, and a stable functional core of microbiome.

Large shifts in the ratio between the five predominantly phyla of bacteria of the gut microbiota or the rising of new bacterial groups determine a disease-promoting imbalance, referred to as dysbiosis. The cardinal features of dysbiosis are the reduction of microbial richness and diversity and outgrowth of Gram-negative lipopolysaccharide (LPS) producing *Proteobacteria* [9].

Dysbiosis is usually characterized by an augmented intestinal permeability [9]. In physiological conditions, the translocation of small number of bacterial products, like polysaccharides of *Bacteroides* spp. or mucosa-adherent segmented filamentous bacteria (SFB), is removed by Th1 and Th17 cells’ action. On the contrary, high numbers of invading bacteria exert an overactivation of Toll-like receptors (TLRs), leading to the overexpression of inflammatory cytokines, with consequent epithelial damage and chronic inflammation [10]. Strikingly, higher SFB levels, as observed in MyD88 (an adaptor for different innate immune receptors which recognize microbial stimuli)-deficient mice models, protect mice of a diabetic genotype from the development of the disease, showing that the microbiota can exert both inhibiting and promoting effects [10]. While a dysbiotic gut community could be the hallmark of several inflammatory diseases, the dysbiosis itself can be trigger for the unbalancing of the intestinal homeostasis and the development of inflammation [10].

It has been associated both with the development and with the severity of an increasing number of diseases, such as Inflammatory Bowel Diseases (IBD), autoimmune disease, obesity, metabolic disease, and neurological disorders. Moreover, it can be the trigger of necrotizing enterocolitis, observed in newborns, or of Clostridium difficile-associated diarrhea, which occurs mostly in elderly people [9].

Unlike infectious diseases, in the gut microbiota, the pathogenicity of specific intestinal bacteria cannot be established by Koch’s postulates’ application, since a major fraction of the gut microbiota cannot be isolated as pure culture. Indeed, microbiota is usually approached with high-throughput DNA sequencing of conserved 16S rRNA genes, or with high-throughput sequencing of all extracted microbial DNA (whole-metagenome shotgun), providing also information about encoded functions of the sequenced microbial DNA [11]. Therefore, the pathogenic implication of specific microorganisms in the development of a pathology relies mainly on the identification of shifted bacterial population or in the replication of a disease through the transplantation of fecal microbiota from an affected mouse to a healthy one.

### The interplay between the gut microbiota and the intestinal barrier

The microbial density increases from the intestinal epithelial cells toward the gut lumen, with the highest number in the latter. The mucosal side is enriched in *Lachnospiraceae, Bifidobacterium bifidum, Bifidobacterium longum, Ruminococcaceae,* and the phylum Verrucomicrobia (represented by the mucus specialist *Akkermansia muciniphila*). Despite the previous hypothesis that the inner mucus layer in the colon is devoid of bacteria in healthy subjects, it has been shown that Acinetobacter spp and Proteobacteria are able to associate with the colonic crypt [7].

The gut microbiota composition influences properties of the mucus layer. In an elegant study comparing genetically identical mice housed in different rooms, the authors discovered that one colony had a colonic mucus layer that was impenetrable by bacteria, while the other colony had the opposite. They suggested that some bacteria, such as Erysipelotrichi class, *Allobaculum* can induce a non-penetrable inner mucus, while other phyla, like TM7 and Proteobacteria have opposite effects [12].

*Bifidobacteria* have demonstrated to reduce inflammation in different in vivo models. They enhance barrier function stabilizing claudins 2 and 4 and occludin at tight junctions in mice models of necrotizing enterocolitis. They also exert antioxidant proprieties and are involved in the maintenance of the intestinal microvilli integrity, in the promotion of anti-inflammatory cytokines production, in the IgA secretion stimulation, and in the maturation of immune cells [13].

A multi-strain probiotic formulation of L. rhamnosus LR 32, B. lactis BL 04, and B. longum BB 536 demonstrated to modulate the expression of TJPs, with the increase of zonulin-1 and 2, occluding and claudin-1, and to prevent inflammatory damage, in an in vitro model of intestinal barrier developed using Caco-2 cell monolayer [14]. Bifidobacterium bifidum ATCC 29521 administration showed to restore the dextran sodium sulfate (DSS)-caused intestinal damage through the restoration of dysbiosis and the regulation of the expression of immune markers and TJs in the colon. Particularly, it determined the upregulation of ROS-scavenging enzymes, anti-inflammatory cytokines, (IL-10, IL-6 and PPAR γ), tight junctions proteins, such as ZO-1, MUC-2, claudin-3, and the downregulation of inflammatory genes (TNF-α, IL-1β) [14].

On the other hand, certain Lactobacillus species prevented barrier disruption through the upregulation of tight junction proteins. In an in vitro model of Caco-2 cells, Lactobacillus rhamnosus GG (LGG) inoculation after INF-γ and TNF-α cells’ stimulation determined the maintenance of transepithelial electrical resistance and ZO-1 distribution in epithelial cells; this effect was partly determined by the inhibition of NF-κB signaling mediated by LGG. Lactobacillus rhamnosus CNCM I-3690 partially restored the function of the intestinal barrier, increased the levels of Occludin and E-cadherin, and reduced bacterial translocation [15].

Different strains of *E*. *coli* have opposite effects on the intestinal barrier; indeed, *E. coli* strain C25 increases intestinal permeability, while *E. coli Nissle 191*7 stimulates Zonulin-2 (ZO-2), and ZO-1 expression, and confers protection against DSS-colitis-associated increase of mucosal permeability to luminal substances in mice models [16].

*Faecalibacterium prausnitzii* has a role in the maintaining of intestinal barrier integrity, through the promotion of the TJ synthesis, ZO-1 expression, and colon epithelial-cell proliferation [15].

The gut microbiota exerts syntrophic, symbiotic, and mutualistic interactions in the mucus layer, thus creating the environment which drives microbial community selection and defines physical properties of the mucus layer.

As previously explained, the mucus layer serves as a source of energy and carbon for mucus residing bacteria.

The so-called “mucus-associated microorganisms”(MAMs) can live, thanks to the presence of both secreted and transmembrane mucin glycans, which serve as attachment sites to glycan-binding components of microorganisms, thus influencing the composition of MAMs [17]. These bacteria, residing in a glycan-rich environment, are able to digest mucus through mucus-degrading enzymes, such as sulphatase, glycosidase, neuraminidase, galactosidase, cysteine protease, and sialidases that cleave the mucus, producing short-chain fatty acids (SCFAs). They are then absorbed and used by colonocytes to recover part of the energy used for the expensive synthesis and secretion of mucin [18]. Within the mucus, there is a wide range of mucus-degrading bacteria: *Bacteroides thetaiotaomicron, Akkermansia muciniphila, Bacteroides fragilis, Bifidobacterium bifidium,* and *Ruminoccous gnavus*. These species can cleave mucus O-glycans to produce monosaccharides, which, in turn, are utilized by other residing bacteria, such as *Clostridium cluster XIV, Lachnospiraceae, Enterobacteriaceae,* and *Clostridium difficile*. *Lactobacillus* and *Bacteroides* showed further adaptation through the presence of multi-repeat cell-surface adhesins which enable retention of the bacteria within the mucus layer. Some mucus residing bacteria may also form mucosal biofilm, complex microbial communities embedded in a polymeric matrix, which can be observed with electronic microscopic in healthy colon of mice, humans, and rats. While using glycans as an energy source, these enzymes produce short-chain fatty acids (SCFAs) through the fermentation process [18]. Moreover, *Akkermansia muciniphila*, representing the 3–5% of the microbiota in healthy individuals, improves the intestinal barrier integrity by stimulating mucin production and complex interaction with gut–bacteria and is associated with low-grade systemic inflammation in human and animal models, insulin sensitivity, and leanness. Indeed, *A. muciniphila*-derived extracellular vesicles increase the expression of tight junctions (TJ) proteins, like occluding (OCLN), thus reducing gut permeability. Other bacteria involved in the maintenance of the intestinal barrier integrity are *Bacteroides vulgatus and Bacteroides dorei*, through the increase of TJ expression and the production of bacteriocins, proteins inhibiting the growth of specific bacteria [19].

### Short-chain fatty acids (SCFA)

Short-chain fatty acids (SCFAs) are products of bacterial metabolism, deriving from the fermentation of indigestible fibers. The 95% of SCFAs in the colon and human feces are represented by butyrate, propionate, and acetate [20]. SCFAs exert multiple physiological functions in the intestine, being involved in the maintenance of homeostasis, induction of epithelial-barrier function and intestinal epithelial cells turnover [20]. They represent an essential energy source for colonocytes and liver gluconeogenesis, also playing a role in the regulation of energy metabolism. SCFA regulate insulin sensitivity and increase the availability of the glucagon-like peptide 1 in the gut. They are involved also in the stimulation of mucin synthesis and in the preservation of gut barrier integrity, inducing TJ assembly. In addition, they exert an immunomodulatory effect through the stimulation of free fatty acid receptors (FFAR); in particular, the four types of FFAR identified are G-protein-coupled receptors (GPCRs), GPR43/FFAR2, GPR41/FFAR3, GPR109A, and Olfr7. These receptors are expressed in several human tissues, such as small bowel, colon, adipose tissue, skeletal, muscle, liver, and pancreatic beta-cells [21]. For instance, butyrate promotes an anti-inflammatory response through the differentiation of Treg cells as well as through the stimulation of nuclear transcription factor, peroxisome proliferator-activated receptor gamma (PPAR-y) which lead to the inhibition of nuclear factor kappa-light-chain-enhancer of activated B cells (NF-kB) pathway and of the activity of histone deacetylases (HDACs). Butyrate also reduces local intestinal inflammation and intestinal permeability through the stimulation of TJ expression and of mucin synthesis. It is the most relevant source of energy for colonocytes and it has a role in their differentiation and proliferation [21].

Microbiota species related to increased short-chain fatty acids (SCFA) production are considered having anti-inflammatory properties: *Lachnospira, Lactobacillus, Akkermansia, Bifidobacterium, Roseburia, Ruminococcus, Faecalibacterium, Clostridium,* and *Dorea* [21].

### Lipopolysaccharide (LPS), TLR4, and NF-κB pathway

The Lipopolysaccharide (LPS) is a component of the Gram-negative bacterial outer membrane with pro-inflammatory properties. It has been identified as a key contributing factor in the initiation and progression of low-grade systemic inflammation [22]. Despite every surface of the human body is colonized by commensal bacteria, the vast majority of microbes reside in the intestine and is composed by Gram-negative bacteria with LPS in their cell wall. Therefore, high levels of LPS often reflect translocation of Gram-negative bacteria from the gut to the blood stream and the interior of the body.

LPS itself has multiple adverse effects on the gut function, promoting intestinal inflammation and disrupting tight junctions (TJ) organization via specific signaling pathways which determines directly enterocytes shedding without compensatory TJ- releasing, and induces oxidative stress in epithelial cells, mitochondrial failure, and mitophagy [22].

LPS indirect action involves the Toll-Like Receptor 4 (TLR4)-Cluster of Differentiations 14 (CD14)-dependent pro-inflammatory response. TLR4 belong to the family of patter recognition receptors (PRRs) and is expressed in several immune cells, such as monocytes, macrophages, and Kupffer cells, but also endothelial cells, adipocytes, and hepatocytes. LPS recognition by TLR4 is helped by LPS-binding protein (LBP) and CD14, with an indispensable contribution of the MD-2 protein stably associated with the extracellular fragment of the receptor [23]. The activation of TLR4 triggers two signaling pathways: the first one involving TIRAP and MyD88 adaptor proteins begins in the plasma membrane, while the second, depending on TRAM and TRIF, is induced in early endosomes after endocytosis of the receptor governed by a GPI-anchored protein, CD14 [23]. Therefore, LPS-induced systemic inflammation is strictly dependent on the rate of TLR4 endocytosis and trafficking through the endo-lysosomal compartment. Usually, MyD88- and TRIF-dependent signaling pathways are triggered consecutively, due to the redistribution of the LPS-activated TLR4 from plasma membrane to the endosomes, leading to its lysosomal degradation and termination of the inflammatory response [24].

These signaling pathways lead to the production of different sets of pro-inflammatory cytokines, respectively, via NF-κB transcription factor pathway activation, and via MAP kinases phosphorylation and subsequent activation of type I PI3-kinase/Akt and transcription factors AP-1 and CREB [24].

TLR4 can be stimulated also by saturated fatty acids (SFA), showing an important role of high-fat diet itself in determining systemic inflammation. This binding could depend on a structural similarity between dietary SFA and the Lipid A of LPS; indeed, SFA’ pro-inflammatory potency varies according to chain lengths, with lauric acid showing the highest pro-inflammatory activity and the myristic acid and the stearic acid the lowest [24].

### Bile acids (BAs)

Bile Acids (BAs) are another important actor in the modulation of the intestinal permeability and, thus, indirectly of low-grade systemic inflammation, both with a direct modulation of the intestinal barrier, and influencing the gut microbiota composition. In general, only 5% of primary BAs is not reabsorbed in the gut and undergo biotransformation to secondary Bas [25]. Secondary BAs play a role in the modulation of immune system activity through BAs receptors, such as Takeda G-protein-coupled BAs receptor-1 (TGR5), farnesoid X receptor (FXR), pregnane X receptor (PXR), retinoid-related orphan receptor-γt (RORyt), and Vitamin D receptor (VDR) [25]. For instance, at physiologic relative low concentrations, secondary BAs exert anti-inflammatory properties, inducing a partial transformation from M1 to M2 phenotype of macrophages and inhibiting the release of pro-inflammatory cytokines, such as TNF-α and IL-6 [25].

Usually, under physiological conditions, intestinal epithelial cells are resistant to BAs’ solubilizing effects. Anyway, at higher concentrations, like those deriving from a high-fat diet, primary and secondary BAs can reduce gut barrier integrity with their detergent properties, causing increased intestinal permeability and, thus, oxidative stress, DNA damage, and production of inflammatory cytokines, such as IL-6 and TNF-alpha, which promote inflammation and cause the inactivation of the FXR, thereby powering a pro-inflammatory state in the colon [25].

Two recently discovered derivatives of LCA, 3-oxoLCA and iso-alloLCA, act as T-cell regulators, inhibiting the differentiation of TH17 cells and increasing the differentiation of Treg cells through [25]. A recent study by Paik et al. identified 12 human gut bacterial genera able to convert LCA to 3-oxoLCA and isoLCA: *Adlercreutzia, Bifidobacterium, Enter-ocloster, Clostridium, Collinsella, Eggerthella, Gordonibacter, Monoglobus, Peptoniphilus, Phocea, Raoultibacter, and Mediterraneibacter* [26].

Previous studies demonstrated that FXR knock-out mice models had reduced epithelial barrier integrity compared to wild-type mice, with a higher incidence of bacterial overgrowth. In that regard, recent studies showed that FXR mediates an increased expression of Occludin, ZO-1, and claudin-1, all proteins involved in the formation of tight junctions, as well as defensins, involved in reducing the intestinal bacterial load [25]. Also, TGR5 seems to have an important role in the maintenance of intestinal barrier integrity, since murine models TGR5 deficient developed an increased intestinal permeability, made of an altered molecular architecture of epithelial thigh junctions with an increased expression and an abnormal distribution of zonulin-1 [25].

Enriched bile composition in primary Bas, such as CA and CDCA, is another mechanism that can stimulate intestinal permeability. This altered composition could be the result of gut microbiota dysbiosis, like that observed in IBD patients, and contributes to the development of intestinal inflammation [25].

### Endocannabinoid system, gut peptides, tight junctions, and gut permeability

The endocannabinoid system (EC) is an important contributor to the hedonic regulation of food intake in mammalians. Studies demonstrated that it is also involved in the regulation of glucose and energy metabolism; and it can be tuned up or down by specific gut microbes (such as *Akkermansia muciniphila*) [27]. Indeed, gut dysbiosis, especially related to obesity of high-fat diet, can increase EC activity, thus, determining an increased gut permeability and consequent LPS translocation. Accordingly, Muccioli et al. demonstrated that blockage of the cannabinoid receptor-1 using CB1 antagonists lowered gut permeability and adipogenesis in obese mice, through normalizing Occludin and ZO-1 expression, whereas CB1 stimulation increased permeability markers both in vivo and in vitro [27].

Gut peptides, such as ghrelin, vasoactive intestinal peptide (VIP), cholecystokinin, glucagon-like peptide 1 (GLP-1), and peptide YY (PYY), are conventionally related to appetite regulation and intestinal motility and secretion. Recent studies discovered their role in mucosal immunity tolerance and maintenance of intestinal barrier integrity. These peptides have also been shown to exert anti-inflammatory properties, probably due to the prevention of bacterial translocation in the gut by the increase of TJ expression, the suppression of pro-inflammatory cytokines released by T cells, monocytes, and dendritic cells, and the prevention of macrophage activation and migration [28]. Studies on mice models showed that a high-fat diet correlates with decreased GLP-1 production, reduced GLP-1 receptor sensitivity, impaired ghrelin secretion, and sensitivity, thus determining a diet-induced low-grade organ-specific and systemic inflammation [28].

## The gut immune system

The commensal gut microbiota exerts a symbiotic relationship with the immune system by maintaining a non-inflammatory homeostasis. This state of immune-tolerance derives from multiple mechanisms such as the mucus barrier minimizing the contact between intestinal microbiota and IECs, and the secretion of antimicrobial peptides like lysozyme, defensins, and immunoglobulin A [10]. Despite the absence of inflammation, the immune system constantly modulates and exerts pressure on gut microbiota; indeed, the absence of one of its components, such as immunoglobulin A can lead to the overgrowth of anaerobic bacteria, especially mucosa-adherent segmented filamentous bacteria (SFB) of Firmicutes, or components of the innate immune system [10].

### Gut microbiota and immune system interactions: key role in systemic inflammation

Different studies discovered that the inactivation of an inflammasome’s component, NOD-like receptor family pyrin domain–containing 6 (NLRP6) protein, in mice, determines the expansion of *Prevotella* spp. and *TM7* bacteria [29]. These mice models are more susceptible to dextran sulfate sodium (DSS)-induced colitis and intestinal infections. This increases susceptibility derives from an impaired mucus secretion from goblet cells in NLRP6-deficient mice, determining a reduced mucus layer [29]. Interestingly, it is the result of the dysbiosis itself instead of the NLRP6-deficiency, as it could be transferred in wild-type mice. In physiological conditions, NLRP-6 activation leads to secretion of IL-18 and IL-1β via caspase-1. IL-18 has a bivalent effect in the intestinal mucosa. It acts as a pro-inflammatory cytokine suppressing mucin production by inhibiting goblet cells’ maturation, as occurs in ulcerative colitis patients; in contrast, IL-18 downregulates IL-22 binding protein, thereby inhibiting the capability of IL-22 to induce intestinal tissue repair and expression of antimicrobial proteins [29].

Toll-like receptor 5 (TLR5), the PRR which recognizes flagellin on the epithelial surface, is also involved in the maintenance of gut microbiota homeostasis. TLR-5 signaling stimulates IL-8 and TNFα secretion in epithelial cells and monocytes, and induces expression of IL-22 and Il-17 in the epithelium [30]. Several studies demonstrated that inactivation of TLR5 determines dysbiosis characterized by altered abundances of more that 100 phylotypes and a bloom in Enterobacteriaceae, especially *E. coli,* thus leading to spontaneous colitis and metabolic syndrome including insulin resistance [30]. The causative role of affected microbiota in the development of metabolic syndrome is confirmed by microbial transplantation from TLR5-deficient to wild-type mice.

The NOD2 receptor regulates the commensal gut microbiota, through the restriction of the total number of bacteria and limiting the colonization by pathogens, especially in the terminal ileum [31]. It is expressed in monocytes and Paneth cells and, its polymorphisms are associated with Crohn’s disease [31]. Impaired function of NOD2 determines the reduction of α-defensin expression in Paneth cells; this leads to the increase of Firmicutes/Bacteroidetes ratio. Indeed, α-defensin reduces the abundance of SFB, belonging to the phylum Firmicutes, and decreases the numbers of Th17 cells producing IL-17 in the lamina propria [32]. SFB in the colon play a role in initiating antimicrobial defense promoting the development of Th17 cells; in turn, IL-17 increases α-defensin secretion, which inhibits expansion of SFB. Therefore, deletion of IL-17 receptor exerts the same effect as NOD2-deficiency in promoting intestinal dysbiosis [32].

SFB is also involved in the promotion of maturation of T-regulatory cells (T-reg) They perform a mutualistic interaction with the intestinal microbiota by secreting anti-inflammatory cytokines, such as IL-10 and Transforming Growth Factor -β (TGF-β). In IL-10 (-/-) mice models, there is an increase of Verrucomicrobia, Bacteroidetes, and Proteobacteria, characterized by a significant increase in *E. coli*, particularly *Escherichia coli NC101* [33]. These bacterial alterations lead to increased intestinal inflammation and, even, colon-rectal cancer. On the other hand, specific bacteria strains and species can promote the production of IL-10, thus, ameliorating gut inflammation, for example: Lactobacilli, Bifidobacteria, or *Faecalibacterium prausnitzii*, which is reduced in Crohn’s disease patients [33].

TGF-β is another anti-inflammatory cytokine, produced not only by T-reg cells, but also by dendritic cells (DC), which contribute in the maintenance of the gut microbiota homeostasis. The absence of TGF-β, in fact, alters gut microbiota composition, determining the increase of *Enterobacteriaceae*, especially *E. coli*. The anti-inflammatory effect of the probiotic *Clostridium butyricum* is the result of the induction of TGF-β signaling in DC, which, in turn, induces Treg cells’ generation [34].

The intestinal microbiota exerts also a crucial role in the maturation of the innate immune system, since gut bacteria are a driving force in this process. Indeed, the absence of microbiota determines an impaired function of neutrophils and DC, with reduced killing of pathogens and secretion of Interferon-I (IFN-I) and IL-15 [35]. Without microbiota, even the development of myeloid cells in bone marrow is delayed. This delay affects the capability of facing systemic infections and increases the susceptibility to allergies. The alteration of gut microbiota homeostasis could also impair the immune system function. Indeed, mice treated with antibiotics in their early development show an increased production of IL-4 and an impaired number of Treg cells, being more susceptible to colitis and asthma [35]. Therefore, we understand the bidirectional relationship between gut microbiota and immune system and how the imbalance of their delicate interplay increases the risk for immune-mediated disorders.

**Fig. 2:**
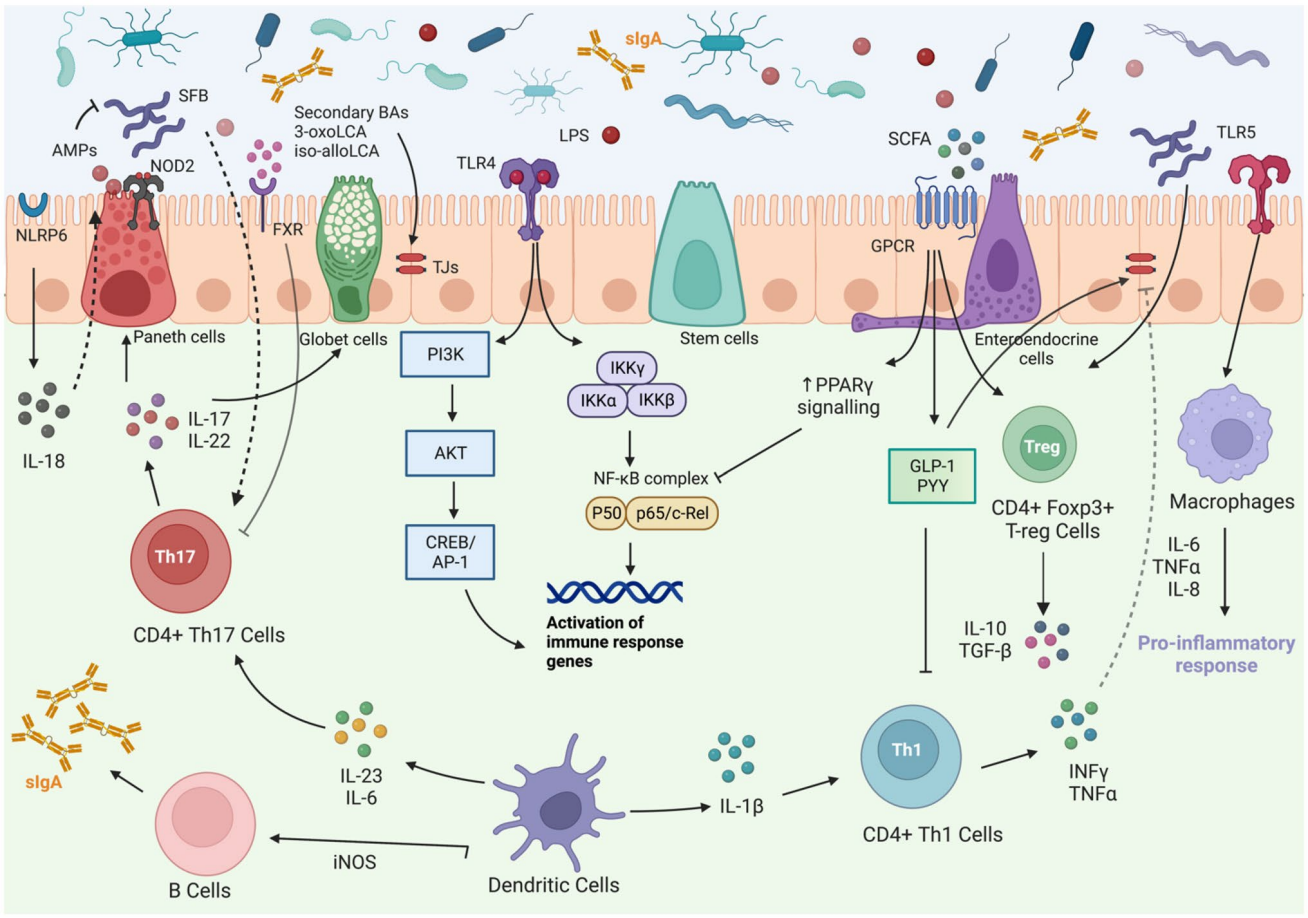
The complex ecosystem of the intestinal barrier. *SCFAs* Short-chain fatty acids; *AMPs* antimicrobial peptides; *sIgA* secretory IgA; *TJs* tight junctions; *IL* Interleukin; *Th* T-helper; *T-reg* T-regulatory; *SFB* segmented filamentous bacteria; *BAs* bile acids; *TLR* Toll-like receptors; *LPS* Lipopolysaccharide; *GPCR* G-protein-coupled receptor; *NLRP6* NOD-like receptor family pyrin domain-containing 6; *iNOS* inducible nitric oxide synthase; *PPAR* peroxisome proliferator-activated receptor; *GLP-1* glucagon-like peptide 1; *PYY* peptide YY; *PI3K* Phosphatidyl inositol 3-Kinase; *NF-kB* Nuclear factor kappa B; *IKK* NF-kB inhibitors

The molecular mechanisms described above are presented in Fig. 2.

## Inflammatory bowel disease

Inflammatory bowel diseases (IBD) are a group of chronic immune-mediated disorders with a relapsing–remitting course that affect the gastrointestinal tract, including ulcerative colitis (UC) and Crohn’s disease (CD). Although the pathogenesis of IBD remains still unclear, their development is considered as the result of genetic, environmental, gut microbiota, intestinal permeability, and immune response factors [36]. Previous studies demonstrated that altered intestinal permeability is present, in asymptomatic patients, years before IBD develops and it is also able to predict future development of the disease [36]. In a study of 1420 first-degree healthy relatives of CD patients, intestinal permeability had been measured using urinary fractional excretion of lactulose/mannitol ratio (LMR). The study demonstrated that the increased intestinal permeability predicts the onset of CD by years; therefore, LMR could represent a possible preclinical marker of the disease. Increased intestinal permeability can be related with the severity of the disease in IBD patients, but usually persists also during periods of remission. In these patients, it is mainly caused by tight junction protein abnormalities, whereas during disease activity, severe mucosal damage disrupts the barrier and causes uncontrolled leakage of the luminal contents [36]. Indeed, TNF-α, together with IL-13, has been implicated in TJ disruption and induction of epithelial-cell apoptosis. Despite its importance, few is known about actors involved in human gut barrier function, since most of the data are limited to animal studies which have shown that microbiota changes can promote disruption of intestinal barrier homeostasis [37].

Patients with IBD are also characterized by gut microbiome dysbiosis. In patients with IBD, it is usually described a reduced bacterial diversity, with a decreased relative abundance of Firmicutes and an increase in pro-inflammatory bacteria, such as Proteobacteria, or adherent-invasive *Escherichia coli* or mucolytic bacteria such as *Ruminococcus gnavus* and *Ruminococcus torques* [37]. The metabolomic approach to the intestinal microbiome suggests that the reduced SCFA levels, caused by the lower abundance of Firmicutes, may have an effect on the immune system. Tryptophan metabolism is also impaired in patients with IBD [38], thus aggravating the severity of the disease; indole has a role too in the maintenance of the gut health [38]. In ulcerative colitis, studies described an alteration of Roseburia and an underrepresentation of *Faecalibacterium prausnitzii*.

Recent studies pointed out the role of bacteriophages and the virome in IBD pathogenesis. A comprehensive virome analysis revealed a reduce core virome in CD patients compared with healthy controls, increased numbers of temperate phage sequences in CD individuals, and changes in virome composition which reflected alterations in bacterial composition in IBD, but no changes in viral richness [39].

Also, the role of the intestinal mycobiota is now under investigation, and the importance of the fungal fraction of the gut could be demonstrated in CD by the biomarker ASCA antibody (anti-Saccharomyces cerevisiae) [38].

Anyway, data presented by studies assessing the microbiome in health and disease are often inconsistent, partly due to variations in microbial composition among different countries, related to culture, ethnicity, alimentary habits, and environmental differences.

In a recent publication, it has been found that decreased prevalence of genus *Adlercreutzia* is associated with impaired barrier function in healthy relatives of CD patients [38]. This taxon has also been described to decrease in subjects who later develop ulcerative colitis and to be inversely correlated, in its relative abundance, with fecal elastase and protease activity before disease onset. Previous studies demonstrated that this genus has anti-inflammatory properties, presumably through its involvement in the metabolism of isoflavones. Moreover, proteases originating from gut bacteria could have a role in the gut barrier function, probably via damage to the gut mucus layer and junction proteins. The same publication also detected an increased abundance of the genus *Colidextribacter* in patients with impaired intestinal permeability [38]: this taxon has previously been described to play a role in the increase of cellular oxidative stress capacity in a mice model, thus contributing to gut barrier impairment [38].

Whether dysbiosis is a cause or a consequence of IBD has not been determined yet. Nevertheless, it has been assessed that pathogenic bacteria can invade the mucosa in IBD patients. Indeed, during active IBDs, the expression of enterotoxigenic Bacteroides fragilis, which is a metalloprotease-producing bacteria, is increased, thus causing inflammatory diarrhea. Moreover, NOD2 mutations are notoriously associated with an increased risk of developing CD; they cause an altered expression of defensin genes, thus facilitating an impaired antimicrobial response to the gut microbiota and promoting the translocation of the bacteria across the epithelium [40].

Recent studies are proposing future IBD diagnostic tools using microbiota analysis. A previous study have identified bacterial markers obtained from *Campylobacter concisus* capable of indicate disease activity in CD [40]. The presence of *Faecalibacterium* is a sign of successful therapy with ustekinumab in patients with CD who developed a primary nonresponse to anti-tumor necrosis factor-alpha (antiTNF-α) [41]. It is crucial to achieve a better understanding of the gut microbiota–intestinal permeability–immune system interaction in IBD to develop novel therapeutic and diagnostic options in the future.

Recently, using mouse models of intestinal inflammation, Carloni et al. showed that during intestinal inflammation, the GVB is disrupted, thus determining a state of bacterial product translocation and systemic inflammation, eventually leading to the propagation of inflammation to the brain, while the vascular barrier in the choroid plexus shuts down, helping protect the brain from inflammation but also potentially impairing some brain functions and causing anxiety [42].

## Gut–liver axis

In 1921, Hoefert, for the first time, described alterations in the gut microbiota of patients with chronic liver disease. Currently, mounting evidences suggest that there is a reciprocal influence between the gut and the liver, known as the “gut–liver axis” [43], since the gut microbiota, microbial metabolites, and reciprocal interaction with the immune system influence the outcome of different liver disease.

The gut–liver axis is the result of a continuous bidirectional “anatomic” communication between the gut and the liver through intestinal blood drained by portal vein, and bile duct, which determines a continuous bidirectional “metabolic” cooperation through biliary acids, hormones, and products of digestion and absorption. The liver is always interacting with the gut-derived bacteria and microbial products, in a distinctive local immune environment, often hesitating in tolerance, since an intact intestinal epithelial barrier can protect the liver from an excess of gut bacteria and their metabolites [44]. As previously discussed, bacterial products, collectively known as PAMPs and MAMPs, derived from the gut, reach the liver via portal circulation and systemic circulation through the mesenteric lymph nodes, and may induce an inflammatory response by activating Toll-like receptors (TLRs). Bacterial translocation can be the original factor triggering liver injury or an additional cause in patients with pre-existing metabolic or viral diseases, particularly those with liver cirrhosis.

Accordingly, studies based on cirrhosis-induced models have shown an early imbalance of the ratio of aerobic/anaerobic bacteria such as the reduction of Gram-positive anaerobic Clostridium clusters. These imbalances were seen to be related to both the increase of pro-inflammatory cytokines (TNF-alpha and IL6) and to the alteration of mechanisms that prevent bacterial translocation, testifying an important role of these microbiological alterations in the evolution of liver disease [44].

The relevance of gut–liver axis has been highlighted also by studies on fecal transplantation (FMT): it has been demonstrated that transferring feces from obese mice with hepatic steatosis, to germ-free mice can reproduce non-alcoholic fatty liver disease (NAFLD) features. Accordingly, it has been described an improvement of intestinal permeability and a consequent therapeutic effect on NAFLD, after fecal microbiota transplantation or after probiotics supplementation [45].

Other interesting observations come from primary sclerosing cholangitis: specific strains of *Klebsiella pneumonia* detected in these patients could damage gut barrier integrity, favoring bacterial translocation of other pathobionts toward the mesenteric lymph nodes, and prime a TH17 cell response in the liver, if transplanted into mice models [46].

### Non-alcoholic fatty liver disease (NAFLD)

One of the most studied is NAFLD and its inflammatory form, non-alcoholic steatohepatitis (NASH). Several studies have reported an influence of intestinal barrier dysfunction in the disease progression. A recent report showed that NAFLD patients with intestinal barrier damage and increased intestinal permeability are characterized by more severe disease status, such as worse liver dysfunction, hyperlipidemia, liver fat deposition, and insulin resistance. Particularly, they found a positive relation between serum D-lactate (a marker of increased intestinal permeability) and markers of hepatocytes’ necrosis, cholestasis, and triglycerides metabolism [47]. Some studies showed a positive correlation between increased intestinal permeability (measured as the lactulose/mannitol ratio), ethanolaemia, endoxaemia, and the degree of liver damage [47].

Probably, it is the result of translocation of bacterial components, particularly LPS, into the portal vein and thus to the liver, resulting in liver inflammation and injury [47]. Moreover, LPS levels seem to be related with fibrosis in NAFLD patients [47].

Some microbial metabolites, such as phenylacetate, had been implicated in women with obesity in lipids accumulation in liver, thus contributing to Non-alcoholic steatohepatitis (NASH) development. At the microbiota level, NAFLD patients, compared with controls, showed an increased abundance of Proteobacteria, Enterobacteriaceae, and Escherichia spp.. Concordantly, children with steatosis or NASH showed depletion in *Oscillospira* spp. accompanied by higher abundance of *Dorea* and *Ruminococcus* spp. [48]. These microbial alterations of metabolic liver disease were associated with increased concentrations of molecules like 2-butanone and 4-methyl-2-pentanone that are responsible of toxic effect in hepatocytes [48].

Other studies have found a positive relation between the fecal microbiome composition and grade of fibrosis in NAFLD: as such an abundance of *Prevotella* has been associated with advanced fibrosis [49]. These findings suggest the possibility to create a fecal microbiome profiling to identify advance fibrosis. Indeed, a diagnostic signature for NAFLD-cirrhosis has been proposed by the combination of microbial, age, and serum measures [49].

In addiction high levels of ethanol-producing bacteria like *E. coli* and *Klebsiella Pneumoniae* have been associated with accelerate NAFLD progression. In this cohort of patients, increased ethanol concentration in blood and breath had been found. These elements support the relation between this condition and systemic inflammation, due to the ethanol-mediated activation of nuclear factor-κB (NF-κB) signaling pathway, which could alter gut barrier integrity, increasing portal and systemic endotoxemia. This way, reactive oxygen species can saturate liver detoxification pathway, resulting in hepatocytes’ damage and hepatic inflammation [49, 50]. Interestingly, ethanol levels in obese patients without NASH are not elevated, further confirming the role of the gut barrier and gut microbiota in metabolic liver disease.

In recent years, growing interest is rising about the evidence of a circulating microbiome signature [50]. As mentioned, gut barrier damage could increase the access of microbial component to the portal flow; consequently, a blood microbiome signature of liver fibrosis in NASH has been described [50]. A recent study analyzed the circulating microbiome of patients with alcoholic hepatitis, and found a decrease in Bacteroidetes and an enrichment of Fusobacteria. These bacteria, characteristics of oral cavity, are associated with increased bacterial virulence and exacerbated endotoxemia. Gut colonization by oral bacteria, especially Klebsiella species, had been responsible for a T-helper 1 (TH1) cell induction and inflammation in genetically susceptible host [46].

Other studies analyzed the circulant microbiome signature in patients with portal hypertension (PH) and cirrhosis. They found a relative increase of some bacteria like *Escherichia, Shigella* and *Prevotella*, which exhibited the higher correlations to IL-8 levels in the hepatic vein. This relation may be induced by the ability of these genera to produce LPS and promote inflammation via toll-like receptor 4 (TLR) or inflammasome cascades [51]. These bacteria were also abundant in patients with model of end-stage liver disease (MELD) scores > 15. Accordingly, it has been assumed that these genera could contribute to the development of cirrhosis and to a pro-inflammatory phenotype of cirrhosis, acting as pro-inflammatory triggers [51].

## Type 2 diabetes mellitus (T2D)

Type 2 diabetes mellitus (T2D) is an acquired condition, characterized by systemic inflammation and increasement of cardiovascular risk and death. The etiology of T2D concerns a combination of multiple gene variations and environmental factors, which are mutual with obesity [52]. Indirect evidences of intestinal hormonal synthesis and microbiota’s contribution to T2D pathogenesis comes from the observed increased risk of T2D in patients with total colectomy [52]. Accordingly, in studies based on germ-free mice, a resistance to diet-induced obesity had been reported; differently, when exposed to bacteria distinctive of obesity (like *Enterobacter cloacae*) or bacteria derived from obese donors, they manifested weight gain and altered glucidic tolerance [52].

T2D had been associated with increased intestinal permeability, able to cause penetration of bacteria across gut barrier, resulting in metabolic endotoxaemia and determining low-grade systemic inflammation [53]. Interestingly, hyperglycemia has been reported to induce an increased intestinal permeability through GLUT2-dependent mechanisms and the alteration of tight junction cohesion, thus creating a leaky gut state [53]. However, it is not totally understood if increased intestinal permeability is a cause or consequence of metabolic disease, or even both. Despite this, gut permeability is becoming an element of increasingly importance in the context of metabolic pathologies. Surprisingly, in a recent study, the predictive role of 4 bacterial species (*Clostridium citroniae C. bolteae, Tyzzerella nexilis, and Ruminococcus gnavus*) had been found over long-term follow-up in type 2 diabetes development. All these bacteria have been associated also with other metabolic diseases and risk factors, such as obesity and inflammatory cytokines secretions [54]. In the past years, different studies had described an altered gut microbiota in T2D, finding a microbial signature for the disease and a relationship between gut microbiota and specific characteristic of T2D such as insulin resistance [54].

Currently, several studies have investigated the role of T2D patients’ microbial signature in influencing gut barrier homeostasis and metabolic endotoxemia. These patients are usually characterized by an increased abundance of Bacteroidetes and Proteobacteria, and a lower abundance of Firmicutes and Bifidobacteria. Not surprisingly, these last genera have been associated with the ability of reducing intestinal permeability, with consequent decrease of endotoxin levels and improvement of glucose tolerance and systemic inflammation [55].

Recent studies conducted on diabetic patients have found a positive relation between some bacterial species and systemic inflammatory markers; in particular, the relative abundance of *Bifidobacterium adolescentis, Alistipes onderdonkii, and Eubacterium rectale* was positively correlated with IL-6, high sensitivity C-reactive protein (Hs-CRP) and LPS-binding protein (LBP), while the abundance of *Bacteroides thetaiotaomicron* was correlated with LBP levels [55]. Furthermore, a member of Firmicutes, *Faecalibacterium prausnitzii,* seems to be reduced in these patients, compared to healthy controls. This bacterium is an important butyrate producer, with demonstrated anti-inflammatory activities, such as expressing tolerogenic cytokines profile to reduce acute, chronic, low-grade inflammation and producing salicylic acid which relates to IL8 level reduction. It could also be involved in maintaining gut barrier functioning, through the synthesis of microbial anti-inflammatory molecules, which regulate cell permeability, preserving tight junction’s proteins, and protecting intestinal cells of the mucous layer [55]. Studies demonstrated that transplantation of this bacteria in diabetic models can improve treatment efficacy and diabetes’ complications [55].

Some bacteria found increased in T2D patients, such as *Prevotella copri* and *Bacteroides vulgatus*, were able to advance insulin resistance and increase availability of branched chain aminoacids in mice. Increased insulin resistance was also shown in mice with diet-induced obesity and treated with *Ralstonia pickettii*, suggesting a potential role of this bacteria in T2D development [56].

However, the multiplicity of pharmacological treatments managed in these patients can modify gut microbiota composition [56]. Nowadays, the research is focused on drug-naive early stages of T2D to better understand the relationship between gut microbiota and T2D. In these drug-naive individuals with prediabetes, the gut microbiota exhibits many early alterations such as a loss of butyrate-producing taxa, an increase of bacteria with pro-inflammatory potentials, as *Ruminococcus* and *Streptococcus*, and a decrease in abundance of *Akkermansia muciniphila*, which shows a potential protective role in T2D development [56].

As mentioned, SCFAs play an important role in glucose homeostasis. It has been reported that a diet abundant in butyrate and acetate is associated with regulatory T cells’ activity improvement, reduced serum levels of diabetogenic cytokines, such as IL-21, and impaired gut barrier integrity [56].

Butyrate appears also involved in reducing the immune response to LPS, helping T-cell differentiations, and decrease IL-6 and IL-12 secretion. Consequently, its deficiency state relates to low-grade inflammation.

Reduced butyrate production in dysbiotic non-obese diabetic (NOD) mice, has been associated with increased colon permeability, increased production of reactive oxygen species (ROS) and pro-inflammatory cytokines like IL-1beta; on the contrary, opposite effects were associated with butyrate intake [57]. In addition to SCFAs, other microbial metabolites are involved in the regulation of carbohydrate metabolism, and they probably play a role in the pathogenesis of diabetes.

Studies showed that the increase of some essential amino acids, such as BCAAs and aromatic amino acids, is connected with a fivefold increased risk of T2DM [57].

Several studies based on obese and diabetic patients have found higher concentration of gut-derived bacteria in blood [57]. Particularly, these models were characterized by increased fasting and post prandial LPS concentration, probably related to gut permeability alteration, which determined the increase of pro-inflammatory cytokines, like interleukin (IL-1) after the infusion [57].

These findings are probably connected with LPS-mediated activation of TLR4 pathways, which stimulate pro-inflammatory signals and modify insulin receptor substrate 1, altering insulin signaling, inducing inflammation, and insulin resistance.

## Type 1 diabetes mellitus (T1D)

Pathogenesis of diabetes has not been completely understood yet, but alterations in gut microbial composition have been described in both type 1 (T1D) and type 2 (T2D) diabetes, suggesting a potential role in the disease development [58].

T1D is characterized by a pro-inflammatory state mediated by pancreatic β-cells, involving both innate and adaptive immunity [58]. Disease development is not entirely explained by genetic predisposition, so other factors have been identified as potential players, such as early exposure to childhood viruses and altered intestinal bacterial composition [58]. Moreover, according to some authors, the clinical onset of T1D is probably preceded by increased intestinal permeability. In this regard, it has been reported that dysfunction of the intestinal barrier with consequent translocation of microbial components through the epithelium and increased presentation of exogenous antigens can stimulate pro-inflammatory pathway activation, not only in the gut and lymph nodes, but also in the pancreas [59]. Interestingly, in T1D patients, alterations of the intestinal mucosal immune system have been described, and mucosal barrier structure and microvilli adhesion are reduced [59]. Indeed, they are characterized by an abundance of IFN-γ-, IL-1α-, and IL-4-producing cells and a reduction of FoxP3 + regulatory T cells (Tregs) [59]. Furthermore, in T1D, lymphocytes directed against specific beta-cell targets express the intestinal α4β7 homing receptor [59]. This suggests an altered lymphocyte homing process, which testifies an initial passage of these cells in the intestine before localizing to the pancreatic islets.

Several studies demonstrate increased paracellular permeability in the small intestine of patients with T1D compared to healthy controls. Accordingly, increased serum markers of intestinal barrier damage and epithelial apoptosis, such as intestinal fatty acid-binding protein (I-FABP), and cytokeratin 18 have been found in patients with T1D [59].

Studies on mice models showed that interventions modifying intestinal permeability in mice can anticipate or delay diabetes onset. In a recent study, treatment of Diabetes prone BioBreeding rats (BBDP) with FZI/0, an antagonist of the zonulin-mediated disruption of tight junctions, decreased intestinal permeability, and reduced the incidence of diabetes. Similar result on diabetes onset were also found after xylooligosaccharide (XOS) administration. Differently, administration of *Citrobacter rodentium*, a bacterium able to disrupt the epithelial barrier, in NOD mice, could increase intestinal permeability, leading to pancreatic insulitis development [60].

Despite large heterogeneity in studies, an altered gut microbiota in T1D patients had been described; particularly, studies based on children with early diagnosis showed increased abundance of Bacteroidetes and Streptococcus mitis, while healthy controls showed higher prevalence of butyrate producers’ bacteria [60]. Nevertheless, opposite result were found in the other reports, in which a high *Firmicutes*/*Bacteroidetes* ratio has been defined as one of the early diagnostic markers of developing autoimmune disorders including T1D. Differences between studies are probably the result of methodological heterogeneity in the research, but they are also the evidence of gut microbiota variability between individuals, probably because of geographical location, ages, gender, type of diet, and medicaments [60]. Despite evidence of connections between diabetes development and intestinal permeability, it is difficult to conclude whether microbial alterations are causal or consequential of T1D and the exact role of gut microbiome in the increase of gut permeability has not been determined yet. Further interventional studies conducted on humans are needed to clear the causal relationship between T1D and intestinal microbiota [60].

## Atherosclerosis and cardiovascular diseases (CAD)

Atherosclerosis is a multifactorial disease, resulting from many risk factors, such as diabetes mellitus, tobacco use, hypertension, and metabolic syndrome [61]. In the atherosclerotic process, alongside these known risk factors, the theory of a gut–systemic circulation axis, characterized by the passage in the bloodstream of bacterially derived products, such as LPS and trimethylamine-N-oxide (TMAO), is increasingly emerging [61].

Previous studies showed an increased intestinal permeability, indirectly evaluated by plasma zonulin measurements, in patients with coronary artery disease or in the acute phase of myocardial infarction. In addition, bacterial DNA was found in human atherosclerotic plaques, especially derived from microorganisms usually present in the oral cavity, and their isolation in fecal samples seems be predictive of coronary heart disease [62]. Interestingly, a pathogenic gut microbiota has been found more frequently in patients with symptomatic atherosclerosis compared to asymptomatics [62].

However, it must be said that the role of the microbiota in increasing the atherogenic process could also derive from its association with previously described metabolic pathologies, constituting a cause for arterial inflammation.

The effect of low-grade endotoxemia on the risk of atherosclerosis has been evaluated in various prospective studies, where significantly increased risk has been demonstrated in patients with high LPS concentrations [62].

Focusing on the role of LPS in atherosclerosis, its traces had been found in macrophages in proximity of arteriosclerotic plaques and not in atherosclerosis-free arteries of the same patient [63]. Higher concentration of LPS had been also found in STEMI patients, but not in stable CAD subjects and healthy controls [63]. In addition, in germ-free mice, a reduction of atherosclerosis has been described, despite high cholesterol levels; this effect seems to be the consequence of the absence of bacteria and the LPS [63].

The pro-atherogenic role of LPS could be explicated by NADPH oxidase 2 (NOX2) activation and ROS production. In some studies, based on the intraperitoneal infusion of LPS in mice models, it was observed an increase in pro-inflammatory cytokines, such as IL8 and TNF alfa, but also the appearance of autoantibodies against ox LDL, the accumulation of inflammatory infiltrate in the vascular intima, and an increasing aortic atherosclerosis [64].

LPS had also been reported to be involved in atherosclerotic plaque vulnerability. Plaque of mice exposed to LPS showed thrombus formation and hemorrhaging phenomena [64]. The report is probably based on the activation of arachidonic acid pathways and synthesis of Leukotriene B4, a strong stimulator of leucocytes activation, whose absence reduces arterial inflammation in mice models [64].

Interestingly, it had been reported a causal role of LPS in the mechanisms of thrombus formation, demonstrating an increase in molecules with prothrombotic affection, such as von Willebrand factor, in human endothelia cells after stimulation with LPS [65]. Furthermore, it was also responsible of in vitro endothelial cells conversion into a pro-inflammatory phenotype, characterized by increased expression of tissue factor, thrombin-activatable fibrinolysis inhibitor and plasminogen activator inhibitor [65]. The increased expression of tissue factor stimulated by LPS seems to be the result of TLR4 activation, since in TLR4 positive endothelial cells, the production of tissue factor was reduced by treatment with TLR4 antibody [65].

Despite large heterogeneity of studies, it has been reported that gut bacteria composition of CAD patients is different from healthy patients. In these patients, it had been described a reduction of overall bacterial richness and evenness [66], with a decrease in Bacteroidetes and Proteobacteria phyla and an increase of the phyla Firmicutes and Fusobacteria [66]. Another study reported a reduction of *Bacteroides vulgatus* and *Bacteroides dorei* in CAD, while, after the administration of these two bacteria in mice, they observed a reduction of atherosclerotic lesion development and improvement of endotoxemia [19].

Relevance of LPS translocation has been studied also in heart failure, where it was associated with worsen cachexia state due to pro-inflammatory cytokines secretion [66]. In these patients, an increased intestinal permeability was found, resulting in increased translocation of bacteria and endotoxins that supply the pro-inflammatory state. Heart failure resulted associated with bacterial overgrowth, shifting to pathogenetic phyla, and decrease in bacteria with anti-inflammatory functions [66].

As mentioned before, other microbial-derived products with a role in atherosclerosis are Trimethylamine-N-Oxide (TMAO). They are produced from elements ingested with the diet, metabolized in Trimethylamine by bacteria, and later in TMAO in liver. Recently, it has been found a positive relation between TMAO concentration and acute coronary syndrome, and therefore, it has been proposed as a predictive and prognostic marker for cardiovascular disease [67]. Furthermore, TMAO levels have been reported to correlate with atherosclerotic plaque dimension and risk for cardiovascular events, such as myocardial infarction, stroke, and death over 3 years [67]. Their levels have also been associated with instability characteristics of the plaque, such as micro-vessels and thinner fibrous cap, through both inflammatory and metabolic pathways [67].

Other bacterial products with a probable implication in this spectrum of diseases are peptidoglycan, SCAFs, and bile acids [68]. Interestingly, Bacterial peptidoglycan has been found in atherosclerotic plaques, and increased expression of genes involved in its synthesis has also been reported in these patients [68]. Peptidoglycan recognition by the immune system is probably mediated by the nucleotide-binding oligomerization domain (NOD) with the consequent alteration of pro-inflammatory pathways such as NF-kB and MAP kinase [68]. Also, bile acids’ metabolism has been described as involved in atherosclerosis development: it is probably related to bacterial bile salt hydrolyzation, which can stimulate enlargement of atherosclerotic plaque through cholesterol accumulation and foam cell formation [68].

Also SCAFs seem to play a role in blood pressure regulation processes and a protective effect in atherosclerosis, stabilizing the plaque. They can regulate renin synthesis via GPR-related pathways, and a hypotensive effect by SCAFs has been described in mice.

## Therapeutic intervention to modulate intestinal permeability

As discussed, intestinal barrier and bacterial-derived products seem to play an increasingly significant role in several chronic diseases. Increased barrier permeability may be the first step in the development of various disorders, not only gastrointestinal disease, or be a cause of their progression; however, there is no gold standard yet for the analysis of barrier function and a clear cause–effect relation has not totally been established.

The role of gut microbiota in these processes is continuously being studied, to find useful information for diagnostic and therapeutic purposes.

In the management and prevention of metabolic endotoxemia and impaired intestinal barrier, a primary role is given to diet. Indeed, habits, such as alcohol abuse, increased consumption of saturated fat acids, or micronutrient-poor diets, contribute to the development of endotoxemia and chronic low-grade inflammation. Moreover, food additives, such as sugar, surfactants, and sodium chloride, have been shown to increase intestinal permeability. In contrast, the use of oils rich in n-3 polyunsaturated fatty acids attenuate the process. Indeed, extra-virgin olive oil (EVOO) has recently been associated with a reduction in postprandial glycemia by improving gut permeability-derived low-grade endotoxemia [69].

Besides diet, there is currently great interest in the use of probiotics and prebiotics, such as Lactobacillus plantarum MB452 or Lactobacillus rhamnosus GG, which have been shown to improve intestinal epithelium survival and cell junction expression, while administration of Bifidobacterum infantis has been correlated with reduced serum endotoxins in mice [15].

In this context, through studies on microbiota in the state of health and disease, new probiotics based on specific commensals with different anti-inflammatory effects, such as *Akkermansia* or *Fecalibacterium,* are being developed [70].

Polysaccharides from Enteromorpha prolifera (EP) are used in the management of obesity and associated metabolic disease. A study by T. Zou et al. demonstrated that EP administration reduce adiposity, insulin resistance, hepatic steatosis, and elevation of serum lipopolysaccharides. Moreover, EP supplementation ameliorated gut dysbiosis induced by high-fat diet, through the increase of short-chain fatty acid (SCFA)-producing bacteria and gut-barrier-protective microbe, and the reduction of endotoxin-producing bacteria [70].

Considering prebiotics, i.e., plant-derived fibers such as Oligo-fructose, Isomaltosextrin or Inulin oligofructose, their supplementation showed beneficial effects in improving gut barrier function, in reducing circulating endotoxin levels, in improving glycaemic status and lipid profiles, both in mice and human models [71].

Beneficial effects have been observed through the supplementation of vitamins, such as Vitamin D, particularly for increasing the richness and diversity of the microbial population and increasing the synthesis of cell junction proteins. In addition, Vitamin A supplementation appears to show beneficial effects on the composition of intestinal bacteria and on reduction of intestinal permeability by increasing the synthesis of TJ proteins. Furthermore, a relevant role in intestinal barrier protection and functioning is achieved by zinc; indeed, its depletion has been associated with increased gut permeability [72].

Another emerging strategy is the production of synbiotics, i.e., the combination of prebiotics and probiotics, such as Bifidobacterium lactis plus fructo-oligosaccharides, which appear to offer a positive contribution in improving intestinal function and decreasing pro-inflammatory cytokines levels, more than the individual elements [73].

Interestingly, confirming the key role of intestinal barrier damage in metabolic disease, positive results come from the use of Lubiprostone (LUB) in NAFLD: it is a bicyclic fatty acid, usually used in constipation, which has the ability to promote intestinal fluid secretion. Clinical trials on NAFLD patients demonstrated that LUB can improve intestinal permeability and reduce levels of hepatocytonecrosis enzymes and blood endotoxins concentration [74].

In the field of liver diseases, several studies tested the role of probiotics in liver cirrhosis, aiming at prevention of cirrhosis complications and infections, and reduction of systemic inflammation through the modulation of gut microbiota and intestinal permeability. A meta-analysis by Saab et al. evaluated the efficacy of probiotics in the management of minimal hepatic encephalopathy (MHE) and overt HE (OHE) in comparison to placebo and lactulose. Overall, they found that the use of probiotics, similarly to lactulose, was more effective in decreasing the hospitalization rates, improving, and preventing the progression to OHE in patients with MHE compared to placebo, but probiotics did not affect mortality rates [75]. A combination of probiotics and prebiotics demonstrated to reduce the rate of infection after liver transplantation if administrated before or on the day of surgery. Moreover, these agents also reduced the total amount of time spent in the hospital or intensive-care unit and the duration of antibiotic therapy use [75].

Immune dysfunction is a common complication of cirrhosis. Horvath et al. tested the effects of a multispecies probiotic on innate immune function, bacterial translocation, and intestinal permeability in a randomized placebo-controlled clinical trial in patients with cirrhosis and demonstrated an increase in serum neopterin levels and production of ROS by neutrophils, and some improvement in liver function in patients treated with probiotics. These results may explain the beneficial effects of probiotics in immune functions [76].

Therapeutic strategies currently under investigation are based on preserving the intestinal barrier integrity and antagonizing the effects of LPS pathways.

Concerning the first target, currently, no specific drug has been approved. Interesting proposals, which could be developed in the future, are epithelial regeneration via stem cells or epithelial growth pathways (EGFR), or the enhancement of tight junctions’ integrity via inhibition of myosin light-chain kinase (MLCK), whose activity correlates with postprandial intestinal permeability. For example, Divertin is a novel molecule that blocks the activity of the mitochondrial hydroxylase (MCLK1) on the peri-junctional actomyosin ring (PAMR) of intestinal cells, thereby reducing actomyosin ring contraction and tightening intercellular junctions. Due to its action on intestinal permeability, it had been proposed as a treatment for IBDs [77]. Other approaches under development are the neutralization of LPS and its related pathways. The main studies in this regard arise from sepsis. Among the strategies developed, there are agents capable of neutralizing LPS, such as cationic lipids or cationic proteins, which are effective in binding LPS in compounds that are difficult to separate, useful to evading LPS-mediated immune stimulation; unfortunately, they have not proved effective in treating sepsis [78]. Interesting prospects have also been opened up by the analysis of antimicrobial peptides (AMPs), i.e., agents involved in intestinal innate immune system, which are useful in neutralizing bacteria and interacting with bacterial cell walls or membranes [79]. Indeed, Peptide 19–2.5, a new synthetic AMP, would appear to act as an anti-endotoxin, binding LPS [79]. Other strategies developed have involved LPS-targeted antibodies (the monoclonal antibody HA.1A, or recombinant human activated protein C) or blocking LPS–TLR4 pathways, however, with unsuccessful results [79]. A possible reason for the failure of these approaches is their application in the context of sepsis; whereas their translation into less severe endotoxemia contexts, such as metabolic diseases, could lead to better results. Nevertheless, studies on sepsis have led to improved knowledge useful for the development of future therapeutic targets.

Additionally, through microbial modulation, FMT appears to improve intestinal barrier functioning and reducing intestinal permeability, providing an important contribution in NAFLD/NASH management by decreasing hepatic fat accumulation and inflammation, both in humans and mice [80]. Furthermore, the effects of FMT on the intestinal barrier integrity assume great relevance in the treatment of hepatic encephalopathy, for showing the ability to reduce of both the absorption and production of ammonium [81]. Despite these findings, there are still conflicting data on the safety and poor data on donor selection and recipient characteristics, so further investigation is needed.

In addition, several clinical trials have been conducted to examine the effect of FMT on IBD, and application has been studied both as a treatment to induce remission and to maintain it, with promising but not entirely satisfactory results, especially when considered on large cohorts. Recent interesting findings come from the combination fecal microbiota transplantation with anti-inflammatory diet, from the use of lyophilised oral fecal microbiota transplantation and from development of synthetic microbial communities [82]. However, it is not currently possible to establish a definite conclusion. Furthermore, in these studies, as in metabolic diseases, the donor selection, methods of fecal administration and preparation, are very heterogeneous and the setting of application of this treatment is still unclear.

## Conclusions

Intestinal barrier and bacterial-derived products seem to play an increasingly significant role in several chronic diseases. Increased barrier permeability may be the first step in the development of various disorders, or be a cause of their progression, not only in gastrointestinal disease.

As discussed, several factors can affect this ecosystem, facilitating bacterial translocation and endotoxemia, leading to a systemic inflammatory response.

However, there is no gold standard yet for the analysis of barrier function and a clear cause–effect relation has not totally been established, although the role of gut microbiota in these processes is continuously being studied, and significant information are emerging for diagnostic and therapeutic purposes.

Accordingly, therapeutic approaches based on modulation of the microbiota, such as diet, pre-probiotics, and FMT, are still in their beginning stages, but current evidence appears highly promising.

Finally, the correct definition of targets, dosages, and a better understanding of individual variability could lead to effective and routine early future clinical use.

## Data Availability

Data sharing is not applicable to this article as no new data were created or analyzed in this study.
